# Influenza A Virus Production Following Quality by Design Principles

**DOI:** 10.1002/elsc.70027

**Published:** 2025-04-23

**Authors:** Tilia Zinnecker, Kristin Thiele, Timo Schmidberger, Yvonne Genzel, Udo Reichl

**Affiliations:** ^1^ Max Planck Institute for Dynamics of Complex Technical Systems Magdeburg Germany; ^2^ Sartorius Stedim Cellca GmbH Ulm Germany; ^3^ Sartorius Stedim Biotech GmbH Göttingen Germany; ^4^ Bioprocess Engineering Otto‐von‐Guericke University Magdeburg Germany

**Keywords:** Ambr 15, cell culture, Design of Experiments, influenza A virus, process development, Quality by Design

## Abstract

Establishing manufacturing processes for cell culture‐based pharmaceutical products involves managing multiple parameters that can affect yield and efficiency, as well as process robustness and product quality. Implementing Quality by Design (QbD) principles can support process optimization, while streamlining the chemistry, manufacturing, and control aspects for regulatory approval. In this study, we mimic a QbD approach based on an influenza A virus production process using two clonal suspension Madin‐Darby canine kidney (MDCK) cell lines with distinct characteristics. We performed a quantitative risk assessment including biological and technical parameters to identify the Critical Process Parameters (CPPs). To comprehensively study the effects and interactions of four CPPs, we used an Ambr 15 scale‐down system following a Design of Experiments (DoE) approach. After data analysis and modeling, we obtained design spaces characterized by high robustness with a less than 1% risk of failure and even some indications for virus titer and yield improvement, while keeping process‐related impurities such as DNA and total protein concentration low. These findings were subsequently verified at a more than 100‐fold higher working volume. Taken together, our approach may stimulate ideas for the implementation of streamlined process development and regulatory approval in the field of viral vaccine production.

**Abbreviations**
CCF: Face‐centered central composite design
CPP: Critical Process Parameter
CQA: Critical Quality Attribute
CSVY: Cell‐specific virus yield
DO
: Dissolved oxygen
DoE: Design of Experiments
EMA: European Medicines Agency
FDA: United States food and drug administration
FMEA: Failure Mode and Effect Analysis
HA: Hemagglutinin assay
HAU: Hemagglutinating unit
IAV: Influenza A virus
ICH: International Council for Harmonization
iVCC: Viable cell concentration at infection time
KPI: Key Performance Indicator
MDCK
: Madin‐Darby canine kidney
MLR: multiple linear regression
MOI: Multiplicity of infection
QbD: Quality by Design
RPN: Risk Priority Number
STR: Stirred‐tank reactor
TCID_50_
: Tissue Culture Infection Dose 50
wv: Working volume

## 1 Introduction

Influenza vaccines are critical for protecting vulnerable populations during seasonal outbreaks and pandemic threats. Although well established, traditional vaccine manufacturing methods including egg‐based production often lack the flexibility and efficiency required to meet the demand in emergency situations. Thus, launching rapid, robust, and scalable manufacturing programs remains crucial. Suspension Madin‐Darby canine kidney (MDCK) cells have been identified as a suitable cell substrate for influenza vaccine production [1, 2, 3]. Yet, process optimization involves navigating several parameters that can influence product yields, efficiency, and product quality. Such parameters can be biological or technical in nature, and their interactions can also affect each other and need to be considered as they ultimately affect safety and regulatory approval. To address these challenges and to synchronize development and approval, Quality by Design (QbD) provides a structured framework for characterizing and optimizing pharmaceutical processes [4]. These principles are rooted in the International Council for Harmonization (ICH) guidelines Q8 to Q11 and are recognized by regulatory authorities such as United States food and drug administration (FDA) and European Medicines Agency (EMA). The main idea is to build quality into every step of the development and manufacturing process, rather than relying solely on testing the final product to ensure it meets the required standards.

**Summary**

- Critical Process Parameters of an influenza virus production process using two clonal suspension Madin‐Darby canine kidney (MDCK) cell lines were identified through quantitative risk assessment.
- Robust processes with a failure rate of less than 1% were obtained for both cell lines from modeling of Design of Experiments cultivation data.
- Slight improvements in titer and yield after scale‐up to more than 100 times the working volume were achieved, while maintaining low levels of process‐related impurities.
- The highly characterized process provides valuable insights that reduce the risk of costly batch failures to ensure a consistent vaccine supply.
- These methods could be adapted to support other cell culture‐based vaccine processes, making them highly relevant for both academic research and industrial manufacturing.

A cornerstone of QbD is the use of Design of Experiments (DoE), a structured approach to exploring the relationships between Critical Process Parameters (CPPs) and Critical Quality Attributes (CQAs). By systematically varying input parameters, a DoE approach can be used to generate design spaces, identify optimal operating conditions, and evaluate robustness. As a result, the process becomes highly characterized and the outcomes of the DoE approach can even be used for further process optimization. Academic studies such as those by Nie et al. have highlighted the value of DoE for the process development of a recombinant adenovirus zoster vaccine and for a live attenuated pseudorabies virus vaccine [5, 6]. Further research on the implementation of QbD principles for vaccines is available, but the manufacturing of RNA‐based vaccines is substantially different from the production of a cell culture‐based whole virus vaccine [7, 8].

In this present study, we showcase a QbD approach for cell culture‐based virus production with the focus on gaining process understanding and improving virus yields. Using Failure Mode and Effect Analysis (FMEA), we evaluated relevant process parameters to identify CPPs for an influenza A virus (IAV) production process in two clonal suspension MDCK cell lines that differ in cell size, growth, metabolism, and virus production yields [9]. We implemented a DoE approach in an Ambr 15 scale‐down system to study the impact of four CPPs and their interplay with the aim to further optimize the process. The robust and optimized conditions were then validated using a 2 L single‐use stirred tank bioreactor (Biostat System) at more than 100 times the working volume (wv).

## 2 Materials and Methods

### 2.1 Cell Line Cultivation and Infection Conditions

Clonal suspension MDCK C59 and C113 cells (Sartorius, Germany) were maintained as previously described [9]. In brief, cells were cultivated in 4 Cell MDXK CD medium (Sartorius, Germany) supplemented with 8 mM l‐glutamine (Sigma–Aldrich, USA) in nonbaffled shake flasks (#431143, Corning, USA) with 30 mL wv. For this, a Multitron Pro incubator (Infors AG, Switzerland) was operated at 37°C in a 5% CO_2_ atmosphere with a shaking frequency of 120 rpm (50 mm throw). Cells were passaged three times a week and inoculated using viable cell concentrations (VCCs) between 5 and 8 × 10^5^ cells/mL. Cell concentration, diameter, and viability were measured using a Vi‐CELL XR automated cell counter (#731050, Beckman Coulter, USA).

For infection experiments in the Ambr 15 system (Sartorius, Germany), cells were centrifuged (300 × *g*, 5 min, room temperature) and seeded at a VCC of 1–4 × 10^6^ cells/mL (Table 1), in 15 mL infection medium containing 3% TrypLE Select Enzyme (10x, #A1217701, ThermoFisher, Germany). The stirring speed was set to 350 rpm (tip speed of 0.21 m/s) and temperature to 34°C. Other process conditions were set and controlled per Ambr 15 vessel according to the DoE design (Section 2.2). For pH control, CO_2_ sparging and the addition of 1 M sodium bicarbonate (Roth, Germany) was used. If needed, a 3% stock solution of antifoam C (Dow Corning, USA) was added to dissolve bubbles.

**TABLE 1:** Overview of the four Design of Experiments factors used at three different levels.

DoE factor	Unit	Low (−1)	Center (0)	High (+1)
pH	—	7.00	7.25	7.50
DO	%	10	30	50
MOI	TCID_50_/cell	0.0001	0.001	0.01
iVCC	cells/mL	1.0 × 10^6^	2.5 × 10^6^	4.0 × 10^6^

Abbreviations: DO, dissolved oxygen; iVCC, viable cell concentration at time of infection; MOI, multiplicity of infection; pH, pH value.

To validate the DoE results at the 2 L scale, we used a Biostat B system (#BB‐8821050, Sartorius, Germany). Here, cells were centrifuged (300 × *g*, 5 min, room temperature) and seeded at a VCC of 8 × 10^5^ cells/mL into a Univessel SU culture vessel (#DUO002LL‐SS01—V, Sartorius, Germany) at a wv of 800 mL. When a VCC of 4 or 8 × 10^6^ cells/mL was reached, the cells were diluted 1:2 with infection medium. A tip speed of 0.21 m/s was kept constant across scales, which corresponds to a stirring speed of 75 rpm in the Biostat B system [9]. In addition, an air–oxygen mixture was sparged for aeration and dissolved oxygen (DO) was maintained at 40%–50% saturation. Culture temperature was kept at 37°C and pH value was controlled at 7.2 during the cell growth phase; during the virus production phase all cultures were shifted to 34°C and pH was controlled at 7.4–7.5.

All infections were performed with an influenza A/Puerto Rico/8/34 H1N1 (Robert Koch Institute, Germany) that was propagated on adherent MDCK cells (#84121903, ECACC, UK). To achieve the desired multiplicity of infection (MOI), seed virus (stock titer: 9.9×10^7^ Tissue Culture Infection Dose 50 [TCID_50_]/mL) was diluted with phosphate‐buffered saline (Roth, Germany), and the respective volume was added to the cell suspension. Virus samples were collected from the supernatant and centrifuged at 3000 × *g* for 5 min at room temperature to remove cell debris, then aliquoted and stored at −80°C until further analysis.

### 2.2 Sample Analysis and Calculations

Total virus titers of the samples were determined by the hemagglutinating unit (HAU) concentration using an hemagglutinin (HA) assay, that has been previously validated giving a maximum standard deviation of ±0.15 log_10_(HAU/100 µL) [10]. Total DNA concentration was determined using a Quant‐iT PicoGreen dsDNA assay and total protein concentration was measured using a Pierce BCA assay kit (ThermoFisher, Germany) according to the manufacturer's instructions.

The concentration of total virus particles *C*_vir_ in the supernatant and the cell‐specific virus yield (CSVY_HA_) were determined using the following equation:

(1) $$C_{\mathrm{vir}}=C_{\mathrm{ery}}\times 10^{\mathrm{lo}g_{10}\left(\frac{\mathrm{HAU}}{100\;\mu L}\right)}$$

(2) $$\mathrm{CSV}Y_{\mathrm{HA}}=\frac{C_{\mathrm{vir},\max}}{\mathrm{VC}C_{\max}}$$

where VCC_max_ is the maximum viable cell concentration obtained postinfection, *C*_vir,max_ the maximum concentration of virus particles according to the HA assay, and *C*_Ery_ is the concentration of chicken erythrocytes used in the HA assay (2 × 10^7^ cells/mL).

### 2.3 Risk Assessment

**FIGURE 1:**
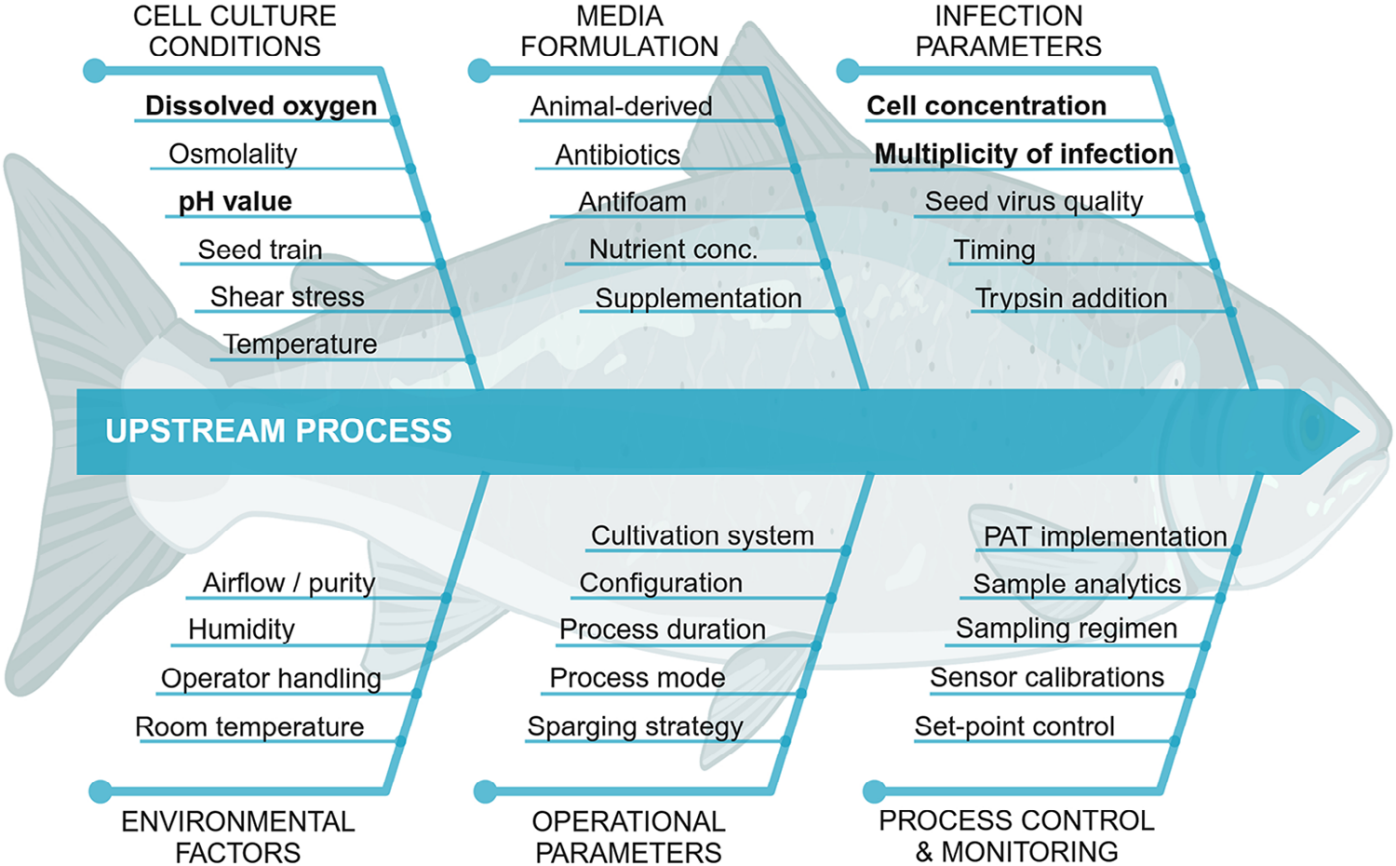
Ishikawa diagram to visualize the root causes of potential problems in a virus production upstream process. Relevant upstream process parameters that may adversely affect the success of an upstream process are organized according to their biological or technical origin. Parameters in bold were identified as critical and therefore included in the Design of Experiments (DoE) to generate a design space. Factors were categorized into specific subgroups and mentioned only once, even if they could be relevant to multiple groups.

Relevant upstream process parameters were mapped in an Ishikawa diagram (Figure 1). An FMEA approach was used for process risk assessment as described by Mollah [11], where the potential risk is divided into three main categories: occurrence, severity, and detection. The occurrence rating assesses the likelihood of a possible failure mode, the failure impact on the production process and the product is assessed by the severity rating, and the possibility to notice and correct a failure mode is assessed by the detection rate. After individually rating each process parameter on a scale between 1 (very low) and 5 (very high), multiplying these ratings results in a Risk Priority Number (RPN) for each respective parameter. An RPN threshold of 15 was used to consider a process parameter as critical and to include it in the DoE experiments for further characterization. To minimize individual bias, the scoring was done by a cross‐functional team from industry and academia.

### 2.4 Design of Experiments

The experimental design and analysis of the results were performed using MODDE 13 software (Sartorius, Germany). A reduced face‐centered central composite design (CCF) was used, including four factors at three different levels that were previously identified by FMEA (Table 1). Center points were included in quadruplicates (*n* = 4) to estimate experimental variability and model reproducibility. This resulted in 24 simultaneous Ambr 15 cultivations per cell line. To improve the comparability of the runs, cells from the same pre‐culture were directly seeded and infected with the respective VCC without a growth phase. pH value and DO were tightly monitored and controlled from the start of the process using the calibrated sensor spots inside the Ambr 15 vessels.

A multiple linear regression (MLR) model was used to fit the model, to determine the design space with a robust setpoint, and to maximize the two DoE responses, total virus titer (HA_max_) and CSVY_HA_, while observing for minimal process‐related impurities given by DNA and protein concentration. We refer to the DoE responses using the terminology of a CQA. However, when considering complete vaccine production, these responses may be understood as Key Performance Indicator (KPI) instead of a CQA, as they are limited to the upstream process. Only model coefficients and interactions considered significant with *p* values <0.05 were included in the model, unless retained for hierarchy in interaction terms. Model statistics were assessed through descriptive power (*R* ^2^), predictive power (*Q* ^2^), as well as model validity and reproducibility.

## 3 Results and Discussion

In this study, we investigated relevant process parameters for IAV production in two clonal suspension MDCK cell lines with distinct characteristics. We evaluated the theoretical risks of each parameter to identify CPPs for further investigation using a DoE methodology in a scale‐down model. We established a design space for each cell line that ensured consistent, high‐titer virus production, and even potential for yield improvement, while keeping process‐related impurities low.

### 3.1 Definition of Critical Quality Attributes Based on Process Development Data

The first step in a QbD approach is initial process development, for which we would like to refer to our previous work [9, 12]. Following an MDCK clone screening in an Ambr 15 system, cell lines C59 and C113 were identified as attractive candidates for a suspension cell‐based influenza virus production process. C59 reached very high cell concentrations of up to 17.3 × 10^6^ cells/mL in batch mode, and after IAV infection maximum titers of 2.97 log_10_(HAU/100 µL) were obtained corresponding to a CSVY of 3900 virions/cell. In contrast, C113 showed lower maximum cell concentrations of 5.5 × 10^6^ cells/mL but very high virus titers of 3.43 log_10_(HAU/100 µL) and a CSVY of 17,771 virions/cell [9]. Based on this knowledge, we used the total virus titer determined by HA assay titer as CQA with a target of 3.0 ± 0.5 log_10_(HAU/100 µL) for C59 and 3.4 ± 0.5 log_10_(HAU/100 µL) for C113, respectively. For vaccines, antigen content and its bioactivity are usually considered as a CQA [13]. However, the amount of HA antigen is usually fixed at 15 µg per strain for an inactivated tri‐ or quadrivalent influenza vaccine, and therefore less useful for characterizing an upstream production process. The use of titer as CQA is described for QbD studies for monoclonal antibody production but was also described for the development of foot‐and‐mouth disease or rabies virus vaccine [6, 14, 15]. The infectious virus titer was additionally tested in our study to serve as CQA, but titers obtained from the TCID_50_ assay did not yield acceptable model statistics in the DoE and were therefore excluded from further analysis (data not shown). Additionally, the CSVY was considered as CQA with the goal of maximizing the value for each of the two cell clones.

We did not evaluate the bioactivity/immunogenicity and other quality attributes because our study is limited to upstream process characterization and does not include a final product intended for regulatory approval, which may even include an adjuvant. Unlike the production of monoclonal antibodies, where CQAs often include glycosylation, charge variants, or aggregation [16], cell‐based viral vaccine production presents its own unique challenges (e.g., the risk of unwanted accumulation of noninfectious virions or defective interfering particles). The process itself defines the product, making it difficult to decouple specific quality attributes from the production conditions [17]. For example, glycosylation is critical to the stability, efficacy, and safety of therapeutic proteins [18]. However, measurement and control are not as well‐established as for therapeutic proteins and its importance in vaccine development has been less considered until recently. To address the challenges associated with process‐related impurities, total protein and DNA concentrations were included in the model. As the observed ranges were not considered problematic (<12 µg/mL) and a high virus yield is key in an upstream process, we used these only as predicted response and not for optimization purposes.

### 3.2 Risk Assessment to Identify Critical Process Parameters

To support a root cause analysis, relevant process parameters that can negatively affect the success of an upstream process for cell culture‐based virus production were assigned to six subgroups of either biological or technical nature and mapped in an Ishikawa diagram (Figure 1). Although we acknowledge the importance of downstream processing and final formulation, this was beyond the scope of our study. Such issues have been covered by other authors, as summarized in a recent review article on QbD considerations for vaccine drying [19].

The parameters listed in the Ishikawa diagram (Figure 1) were then used to conduct a comprehensive FMEA, where we assigned values ranging from 1 to 5 to occurrence, severity, and detection based on our experience with this process. In FMEA, occurrence measures how often a failure is likely to occur, ranging from rare events (1) to frequent problems (5). Severity evaluates the impact of a failure, with low values indicating minor effects and high values indicating serious consequences to the process or product. Detection assesses the likelihood of identifying the failure in time, where a low value reflects a strong ability to notice problems early, while a high value indicates that the failure is difficult to detect, potentially leading to greater risks. Multiplication of the assigned values in these three categories resulted in an RPN and parameters with an RPN greater than or equal to 15 were considered for further analysis in the DoE.

Media formulation in general is highly critical to a successful cell‐based process, as the media provides the essential nutrients for cell growth, should facilitate single‐cell growth, and may support virus production. Certainly, media for cell growth and for virus production may be different. Here, the implementation of DoE can be very useful to develop or improve the media composition [20]. However, all parameters from the media formulation category were excluded from our FMEA, as an appropriate medium was chosen in the initial process development phase and changes were not in the scope of the project [9]. Because academic research laboratories do not provide the tightly controlled and closed environment of a good manufacturing practice facility, environmental factors were also excluded from the FMEA.

The FMEA results for all parameters considered are shown in Table 2. Although the seed train had a high RPN of 16 and was considered critical in comparable QbD studies [21, 22], we decided not to include it in our study. When we investigated stability by culturing cells for over 21 passages, freezing cell banks at Passages 7, 14, and 21 and then testing virus titers for these cell banks after thawing, we obtained very similar titers for all three passages (Figure S1). From this result, we decided to not further consider this as a critical parameter. Thus, FMEA results indicated that pH value, DO concentration, MOI, and viable cell concentration at infection time (iVCC) should be used as potential CPPs for IAV production and in the following DoE. Although the occurrence and detection ratings for pH value were relatively low (2), we assigned a high severity of 5, because pH values below 7 are associated with a significant risk of disrupting viral stability [23]. The DO is also a critical factor, as oxygen availability directly affects cellular metabolism, influencing both cell growth kinetics and viral productivity. The MOI determines the virus‐to‐cell ratio at the time of infection, which can significantly affect infection dynamics, and overall yield. A low MOI can result in suboptimal infection rates, while a high MOI can lead to premature virus‐induced cell lysis or accumulation of defective interfering particles [24, 25]. Similarly, a high iVCC has the potential to enhance virus propagation by providing more host cells for infection. However, excessive VCC may lead to nutrient depletion, accumulation of inhibitory by‐products, or limitation of virus release due to physical constraints. Optimizing iVCC is therefore critical to balancing cell growth and virus production. Consistent with our analysis, these potential CPPs have also been identified in similar studies for cell culture‐based protein or virus production [6, 14, 26].

**TABLE 2:** Failure mode and effect analysis to identify critical process parameters.

Group	Parameter	Occurrence	Severity	Detection	RPN
Cell culture conditions	Dissolved oxygen concentration	2	4	2	16
	Osmolality	2	2	1	4
	pH value	2	5	2	20
	Seed train	2	4	2	16
	Shear stress	1	2	1	2
	Temperature	1	4	2	8
Infection parameters	Cell concentration	3	5	1	15
	Multiplicity of infection	2	3	4	24
	Seed virus quality	1	2	4	8
	Timing	1	5	1	5
	Trypsin concentration	2	1	4	8
Operational parameters	Configuration	1	3	1	3
	Cultivation system	1	1	1	1
	Process duration	2	1	1	2
	Process mode	1	4	1	4
	Sparging strategy	2	4	1	8
Process control and monitoring	PAT implementation	1	4	1	4
	Sample analytics	2	4	1	8
	Sampling regimen	1	1	1	1
	Sensor calibrations	1	3	1	3
	Set‐point control	2	4	1	8

*Note:* Risk priority number (RPN) obtained by multiplying individual risks. A value of 1 represents no potential risk, 3 represents moderate/controllable risk, and 5 represents a significant risk. Parameters with RPNs ≥ 15 were considered as critical process parameters.

Other cell culture and infection parameters such as temperature, trypsin concentration, and sparging strategy were recognized as influential variables, but generally less critical, resulting in lower RPN scores. Seed virus quality was assigned a relatively low severity value in this study because the primary focus for inactivated influenza vaccine production is the total virus particle yield rather than the proportion of infectious particles. However, for other applications, such as adeno‐associated virus production, where the presence of empty capsids has a significant impact on product quality and therapeutic efficacy, seed quality would be considered more critical and assigned a higher severity value. Moreover, shear stress may be a critical factor for more sensitive cultures such as primary cells, hybridoma cells, or adherent cells on microcarriers. However, for MDCK suspension cells tested in stirred‐tank reactors (STRs) in a range between 80 and 220 rpm, no severe effects were observed [27, 28].

Control of all incoming raw materials would be important [16] but was neglected in our study because our process is animal‐component free, resulting in less variation compared to processes using, for example, fetal calf serum. In addition, we only had media from one LOT available, and no antibiotics were used. All operational and process control parameters were considered less critical because their occurrence is very unlikely or has minor consequences. The process mode is predetermined during the development stage, and the established parameters were assumed to be maintained within acceptable ranges, thereby minimizing the likelihood of failure modes and associated risks. Moreover, a proper analytical assay validation and calibration strategy is assumed. As a result, these parameters were considered less critical in the context of the FMEA for the influenza virus manufacturing process.

### 3.3 DoE Set‐Up and Data Modeling of Cultivation Data

To investigate the four identified CPPs at three levels, we used a reduced CCF design with 20 experimental conditions and additionally, four center point replicates to ensure robust data. The choice of a CCF design over a full factorial design drastically reduced the number of experiments required while characterizing the potential CPPs well. However, an appropriate scale‐down model had to be selected to perform all the planned cultivations, which is also common practice in the industry [29]. We used an Ambr 15 system, which allows to run 24 cultivations in parallel with a minimum wv of 10 mL under stirred conditions with pH value and DO control. For better comparability, we decided to skip the cell growth phase in the Ambr 15 system and inoculate the cells directly at the desired iVCC from the same preculture.

MLR was used to model the data collected from the cultivation experiments. The model was optimized by removing insignificant coefficients unless an interaction term was considered significant (*p* value < 0.05), revealing pH value and iVCC as the main drivers of process outcomes. Whether other significant effects were shown, and if so, in what direction, was specific to the CQA and also dependent on the cell line. All included model coefficients for both cell lines and all CQAs are shown in Figure S2.

Various statistical metrics were evaluated to assess the fit and applicability of the model (Figure S3). The descriptive power (*R* ^2^) should be greater than 0.5 for a high model significance, while the predictive power (*Q* ^2^) should be at least a value of 0.1, but ideally greater than 0.5. The difference between the two metrics should not be bigger than 0.3. Such values were achieved and even exceeded for both cell lines and all four CQAs, except for the total protein concentration in C113, which showed a low *R* ^2^ of 0.31 and *Q* ^2^ of 0.15. Since the total protein concentration was only used for approximate prediction and not for optimization, we decided to keep the data in the model. The model validity statistic is evaluated to identify potential problems, such as outliers, incorrect model terms, or transformation errors. A validity score below 0.25 indicates the presence of statistically significant problems. Reproducibility, on the other hand, measures the consistency of the center point replicates relative to the overall variability and should yield a value above 0.5 for a highly reliable model. Overall, we achieved very high validity and reproducibility metrics, confirming the usefulness of the model.

### 3.4 Infection Cell Concentration and pH Value Mainly Contributed to Process Performance

**FIGURE 2:**
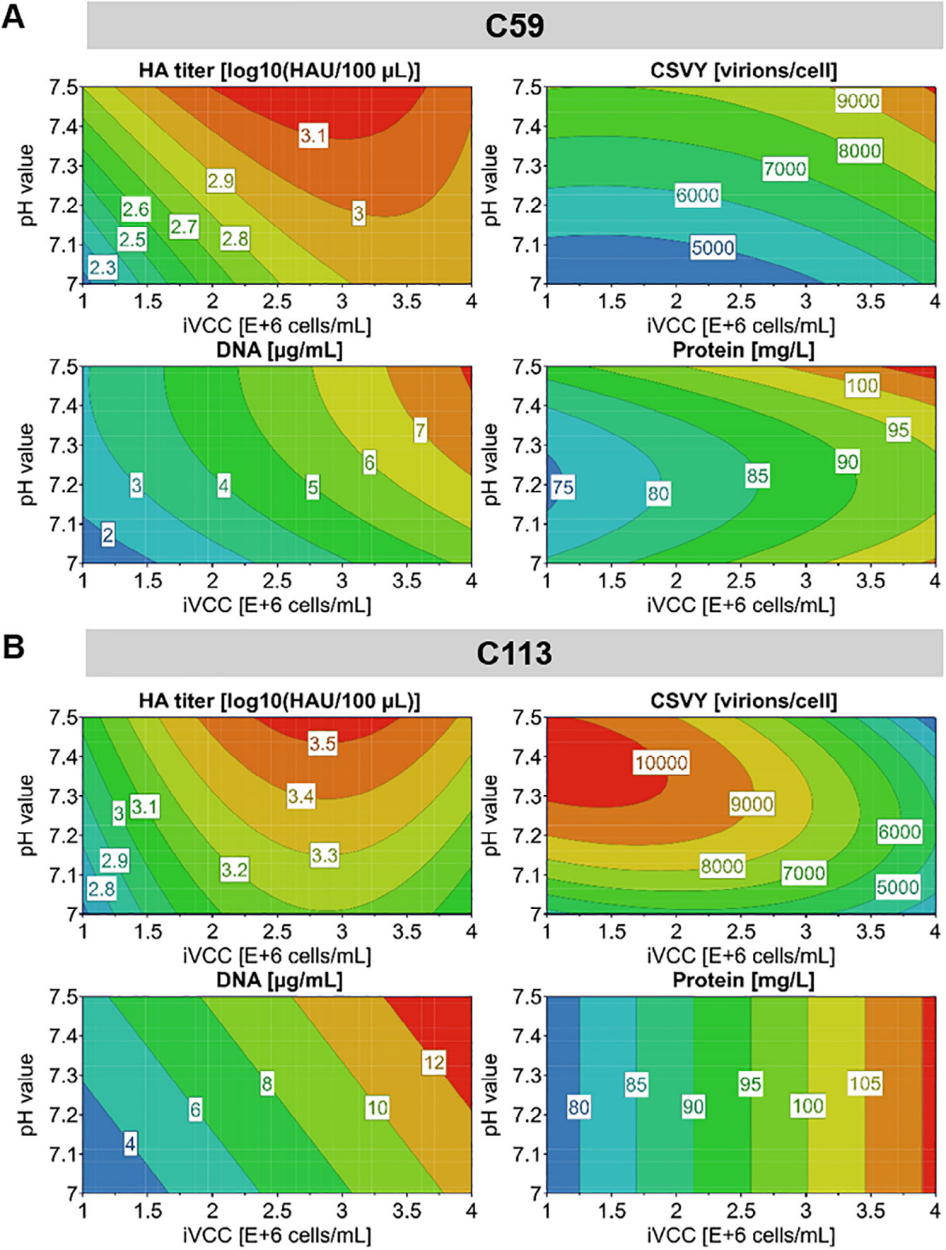
Two‐dimensional contour plot based on the two significant model terms pH value and iVCC. Responses are given for HA titer, CSVY, total DNA, and protein concentration with respect to the cell lines C59 (A) and C113 (B). The numbers in the boxes indicate the predicted values estimated by the model, for HA and CSVY highest values (red) are targeted, for DNA and protein the lowest values (blue) are of interest. CSVY, cell‐specific virus yield; HA, hemagglutinin assay; iVCC, viable cell concentration at infection time.

Following, we used the model to understand the relationships between the tested factors and their interplay with the CQAs of the process. Although DO and MOI were selected as key process parameters (see Section 3.2), no significant effects on HA titer were identified in the DoE for C59. Interestingly, for C113, the DO had a significant effect on the HA titer, while MOI showed no significance but was retained in the model due to a significant interaction term iVCC × MOI (Figure S2). The coefficient plot shows that pH and iVCC are the most influential CPPs with respect to the peak HA titers (Figure S2). To illustrate the model results, we generated two‐dimensional contour plots showing their effects on the CQAs (Figure 2). A higher pH value consistently increased metrics for all CQAs across both cell lines and was thus shown to be favorable for virus production. Generally, a physiological pH value is recommended in the literature for virus production, while pH values below 7 are associated with a risk to disrupt virus stability [23].

**FIGURE 3:**
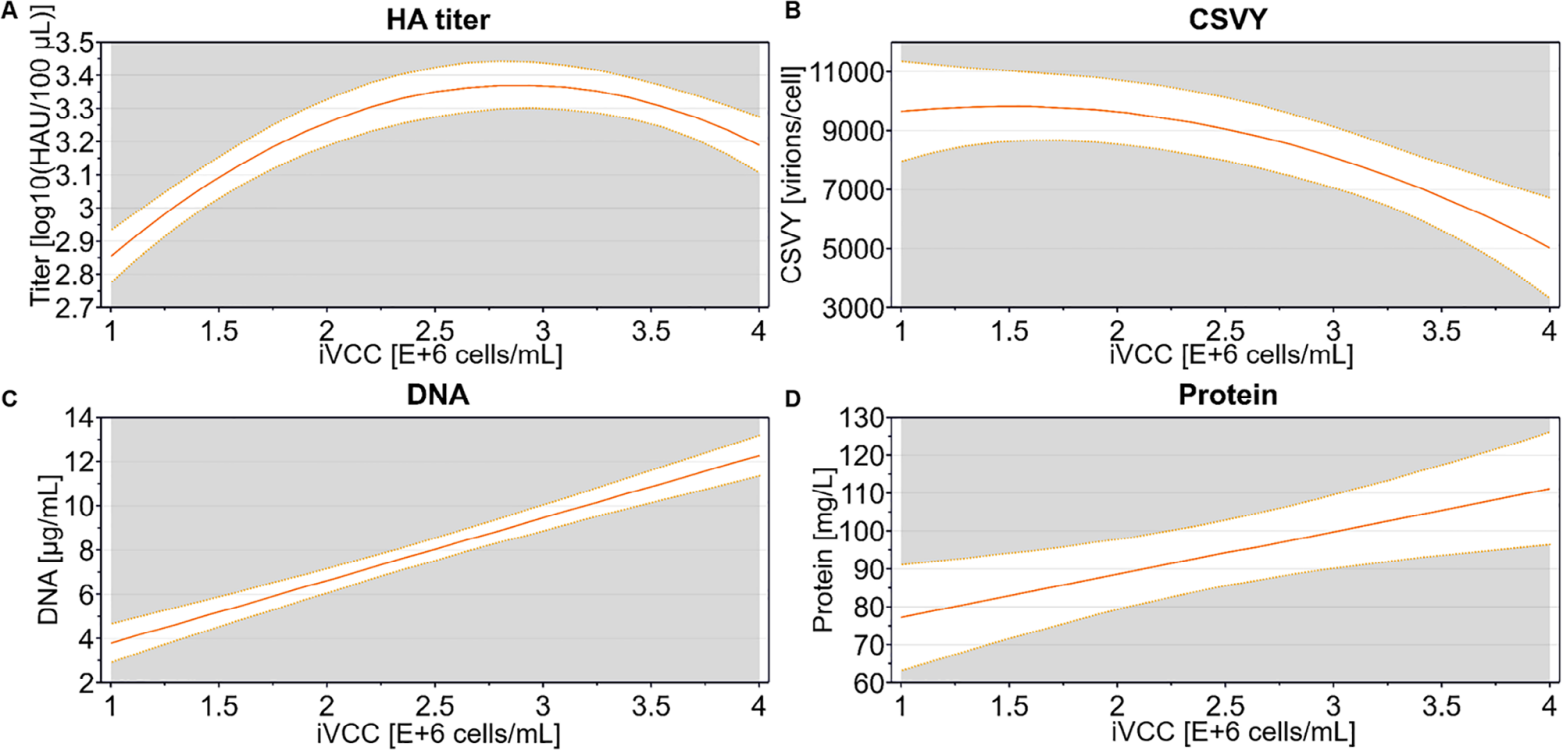
Effect of iVCC on the critical quality attributes for C113. Factor effect plots are given for HA titer, CSVY, total DNA, and protein concentration. Solid lines show the model prediction, while dotted lines show the lower and upper confidence intervals (95%). CSVY, cell‐specific virus yield; HA, hemagglutinin assay; iVCC, viable cell concentration at infection time.

For both cell lines, the model predicted optimal iVCCs between 2 and 3 × 10^6^ cells/mL for achieving high virus titers. However, the effect on CSVY varied between cell lines. A slight increase in iVCC boosted the CSVY for C59 (Figure 2A), whereas for C113, higher iVCC led to a decline in CSVY (Figures 2B and 3). The factor effect plot of C113 shows that the HA titer followed a nonlinear, parabolic trend with iVCC (Figure 3A), indicating that higher cell densities do not always enhance outcomes, probably relating to what is known as the cell density effect [30]. Accordingly, the CSVY also decreased with increasing iVCC (Figure 3B) following a monotonic trend.

As expected, total DNA and protein concentrations increased linearly with iVCC (Figure 3C, D). Yet, the ranges achieved for those impurities were comparable to what has been described previously in the literature before a DNA digestion step [12].

### 3.5 Design Space Exploration to Identify Robust and Optimal Process Setpoints

Process robustness is a hallmark of any industrial process and is even considered to be more important than yield. For both cell lines, highly robust design spaces were obtained, achieving less than 1% risk of process failure. In a recent study by Hood et al., a similar QbD approach was used for CAR‐T cell therapy. Here, high variability is introduced not only by patient material but also by uncontrolled, highly manual processes [22]. Thus, our influenza A virus production process shows superior robustness compared to some other cell culture‐based productions.

**FIGURE 4:**
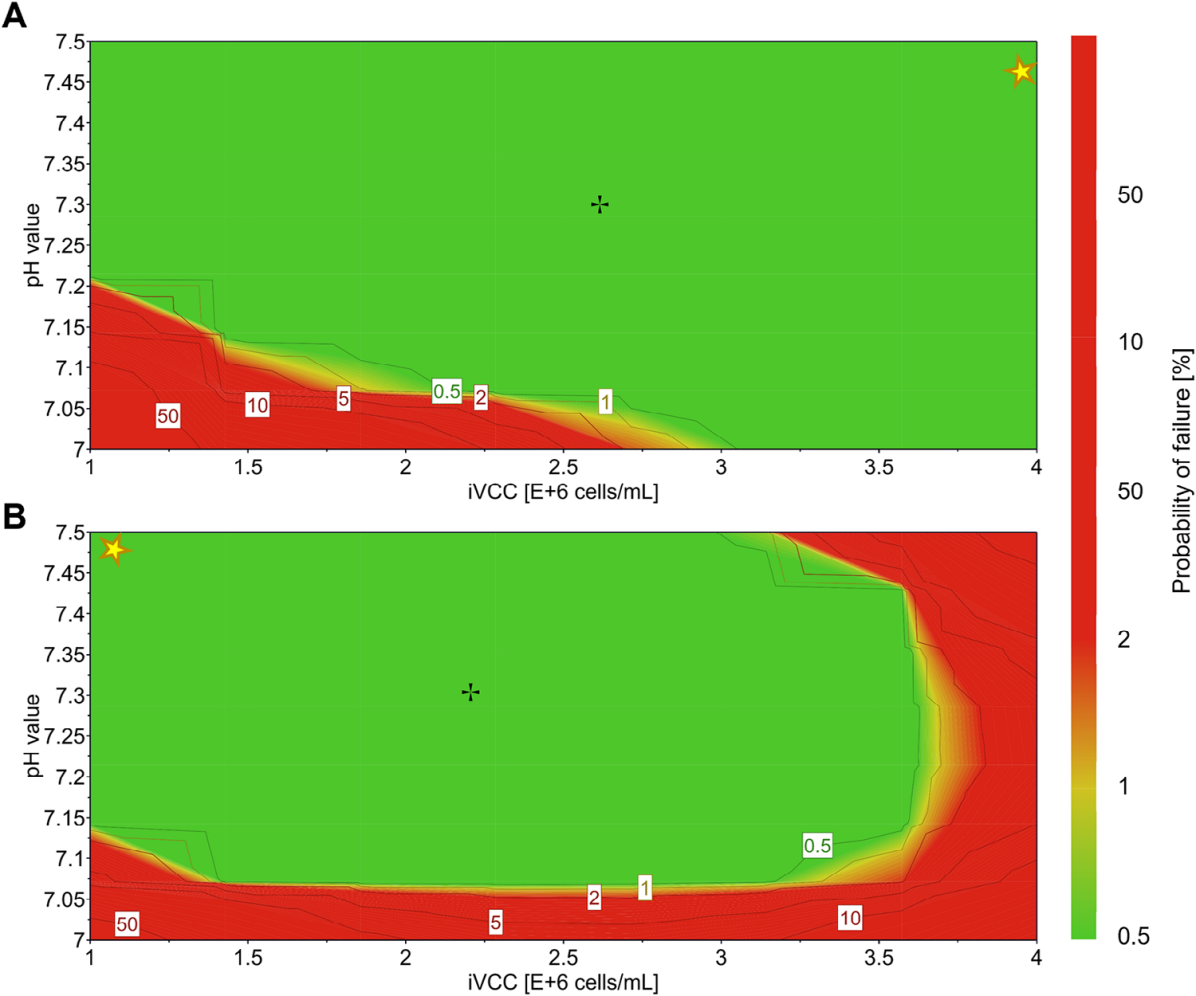
Design space based on a multiple linear regression model for an influenza A virus production process indicates highly robust processes. Design space determined for C59 (A) and C113 (B) based on pH value and iVCC. MOI, pH value, iVCC, and DO were tested according to the CCF design at three levels, center point cultivations were performed with *n* = 4. For C59, DO was set to 40 and MOI to 0.001; for C113, a DO of 50 and MOI of 0.001 was used. The robust setpoint is shown with a black cross, the predicted optimum with a yellow star; the color code and numbers indicate the risk of failure to meet CQA. CCF, face‐centered central composite design; CQA, Critical Quality Attribute; DO, dissolved oxygen; iVCC, viable cell concentration at infection time; MOI, multiplicity of infection.

Looking at the design spaces shown in Figure 4, it may seem that the factor ranges tested have narrowed the potential for observing significant process variation. However, the ranges used in our study were carefully selected based on previous experience and published data with a focus on economically and technically relevant conditions. Moreover, we believe that the ranges are broad enough to capture the measurement and validation errors of the probes used. Various influenza virus production processes described in the literature are operated at a pH value of 7.2 [12, 31, 32]. However, our data modeling suggested that robust processes could be controlled for both cell lines at a slightly higher pH of 7.3 and for optimal conditions even at 7.45 (C59) and 7.5 (C113), respectively (Table 3). Even higher pH values of 7.5–8.0 have been shown to be favorable for optimized production of a herpes simplex virus‐based vector [33].

**TABLE 3:** Model‐predicted values for robust and optimal setpoints of critical process parameters for both cell lines.

	C59		C113	
DoE factor	Robust	Optimum	Robust	Optimum
pH	7.30	7.45	7.30	7.50
DO	40	50	50	40
MOI	0.001	0.001	0.001	0.01
iVCC	2.6 × 10^6^	4.0 × 10^6^	2.2 × 10^6^	1.0 × 10^6^

Abbreviations: DO, dissolved oxygen; iVCC, viable cell concentration at time of infection; MOI, multiplicity of infection; pH, pH value.

As MDCK cells are known to produce metabolic by‐products such as lactate and glutamate during the growth and production phase [9], a combination of reduced CO_2_ sparging and base addition should be considered to maintain these rather high setpoints. Interestingly, iVCCs between 2 and 3 × 10^6^ cells/mL were predicted for both cell lines to achieve a robust process, while for optimal conditions C59 should be infected at 4 × 10^6^ cells/mL and C113 at 1×10^6^ cells/mL, respectively (Table 3). In general, a robust setpoint ensures consistent product quality by remaining resilient to minor process variations, while an optimal setpoint maximizes process performance or efficiency within the acceptable range for CQAs. Setpoints for DO and MOI are also shown in Table 3, but the effect of these CPPs was rather negligible under the conditions tested. Thus, the severity value of these parameters could be reduced in a subsequent FMEA. Since the use of seed virus adds significantly to the cost of a manufacturing process, a low MOI is generally favorable. Although less critical for an inactivated vaccine produced in batch mode, a high MOI also carries the risk of accumulation of defective interfering particles [24]. Taken together, the DoE methodology allowed us to define robust operating ranges for each factor, providing valuable guidance for optimizing virus yield while minimizing variability in the upstream process.

### 3.6 Process Validation and Optimization at the 2 L Scale

**FIGURE 5:**
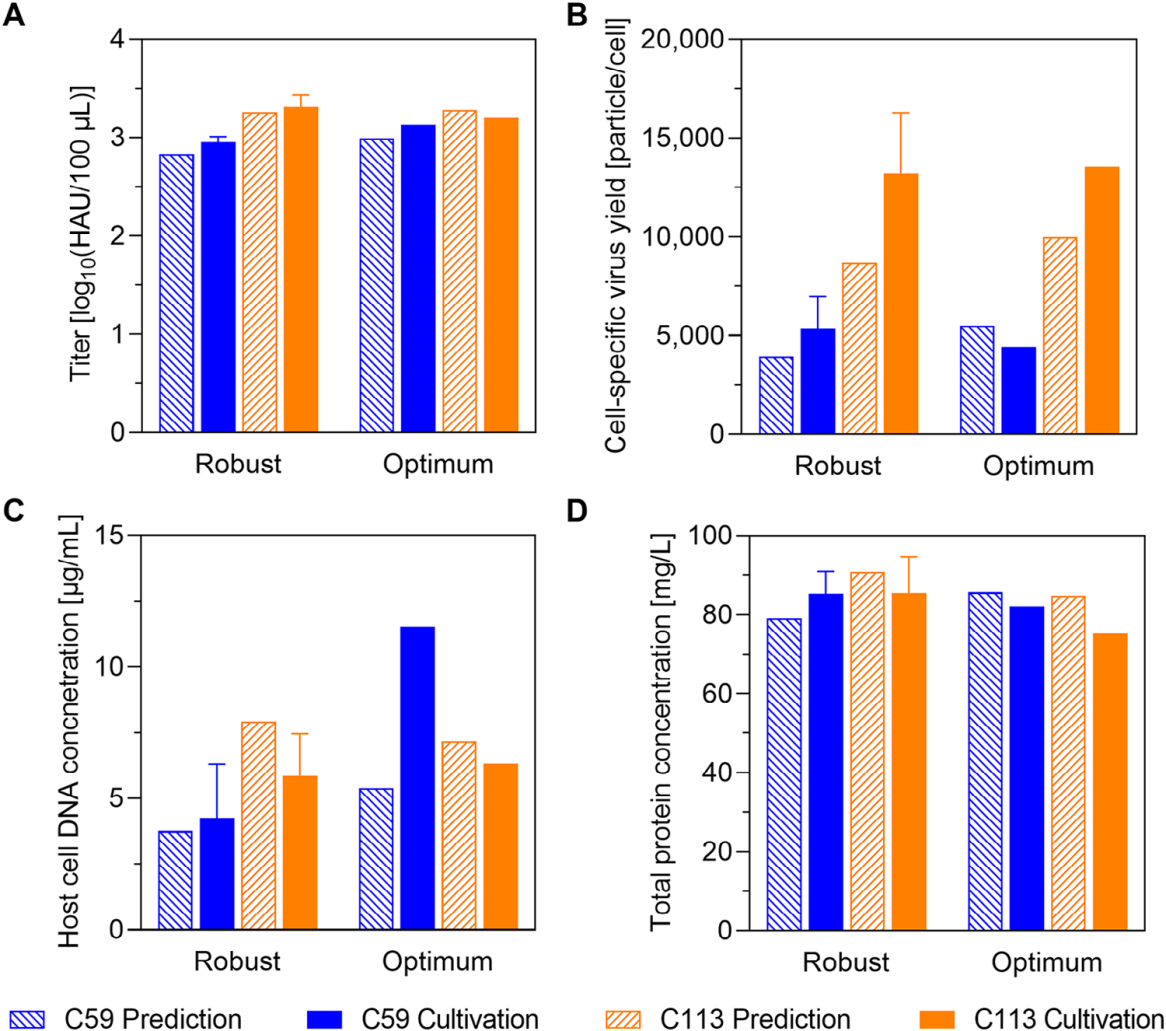
Validation and optimization results for virus production at the 2 L scale. Robust and optimal setpoints obtained from Design of Experiments (DoE) modeling (shaded bars) were verified in 2 L stirred tank bioreactor cultivations (full bars) for both cell lines C59 (blue) and C113 (orange). Total virus titer (A), cell‐specific virus yield (B), total DNA (C), and protein concentration (D) at 3 days postinfection are shown. Cultivations to determine the robust set point were performed in triplicate (*n* = 3, mean ± standard deviation), for the optimum setpoint a single cultivation was done (*n* = 1).

The predictive power of our model was validated at the 2 L scale in a single‐use Biostat STR system, which represents a more than 100‐fold increase in wv compared to the Ambr 15 vessels. The tip speed was chosen as the scaling parameter as previously shown [9, 34]. For both cell lines, the model‐predicted robust setpoints were evaluated in triplicate, while the optimum was tested in a single cultivation (Table 3). Total virus titers, CSVYs, total DNA, and protein concentrations for both robust and optimized conditions were compared with the predictions, providing a comprehensive evaluation of the model (Figure 5).

Titers were successfully reproduced across scales with deviations being smaller than the error of the HA assay for both cell lines. To minimize the number of experiments, we tested the predicted optimum at the edge of the design space directly at the 2 L scale. The optimization attempt for C59 yielded a slight improvement of about 0.23 log in virus titers over the robust setpoints. Notably, total protein concentrations were reduced under optimized conditions, consistent with process goals to minimize impurities. Although DNA concentrations were slightly higher in the optimized run, they remained well below 12 µg/mL, a threshold that we believe is not problematic for an upstream production process and can be effectively addressed in subsequent purification steps. In fact, even higher levels of host cell DNA have been reported for instance for rVSV production in Vero cells, which showed 18.5 µg/mL in the harvested supernatant. However, these levels were efficiently reduced by 99.9% by downstream processing [35]. All predicted values for the total protein concentration were in a comparable range to the cultivation data, although the model statistics for C113 were not ideal. After optimization, the cultivation for C113 did not show any significant improvements, although the same yields were achieved at lower cell concentrations and the experimental results were in line with the model predictions. The scale‐up data validate the DoE model as a tool for optimizing upstream influenza vaccine production and demonstrate its potential to improve titers while keeping impurity levels within acceptable limits.

## 4 Concluding Remarks

Implementing of QbD can accelerate regulatory approval while simultaneously providing a deep understanding of the underlying processes to mitigate potential risks of a costly batch failure. This includes defining an operating range that ensures a successful process with respect to the CQAs that address process efficiency or product quality. Based on an existing upstream process, CPPs were identified and investigated using a DoE in an Ambr 15 scale‐down model. Tightly controlled cultivation followed by data analysis and modeling revealed a highly robust batch process with <1% risk of failure. However, testing a broader range for the CPPs may be relevant to achieve a larger characterized knowledge space for regulatory approval. Individual design spaces were explored for both cell lines with performance variations mainly due to pH and iVCC. The robust setpoints were validated and challenged in a single‐use Biostat STR system at a more than 100‐fold higher wv. Although cell line C113 showed higher yields, C59 may be preferred for an industrial process due to the superior reproducibility of cell growth properties. Taken together, these results pave the way for an industrial‐scale upstream batch process that ensures consistent, high‐quality virus production for inactivated vaccine applications.

## Author Contributions

Conceptualization: Tilia Zinnecker and Yvonne Genzel. Methodology: Tilia Zinnecker, Udo Reichl. Investigation: Tilia Zinnecker. Writing—original draft: Tilia Zinnecker. Writing—review and editing: Tilia Zinnecker, Kristin Thiele, Timo Schmidberger, Udo Reichl, and Yvonne Genzel. Supervision: Udo Reichl and Yvonne Genzel. Project administration: Tilia Zinnecker, Kristin Thiele, and Yvonne Genzel.

## Conflicts of Interest

Yvonne Genzel and Udo Reichl declare no conflict of interest. Tilia Zinnecker was supported by a grant from Sartorius. Kristin Thiele and Timo Schmidberger are employed at Sartorius.

## Supporting information



Supporting Information

## Data Availability

The data that support the findings of this study are available from the corresponding author upon reasonable request.
