# Supplementary material for: Influenza A Virus Production Following Quality by Design Principles
Source: Eng Life Sci. 2025 Apr 23;25(4):e70027. doi: 10.1002/elsc.70027 (PMC12016631; doi:10.1002/elsc.70027)
Supplement: Supplementary file 1 — Supporting Information [file ELSC-25-e70027-s001.docx]

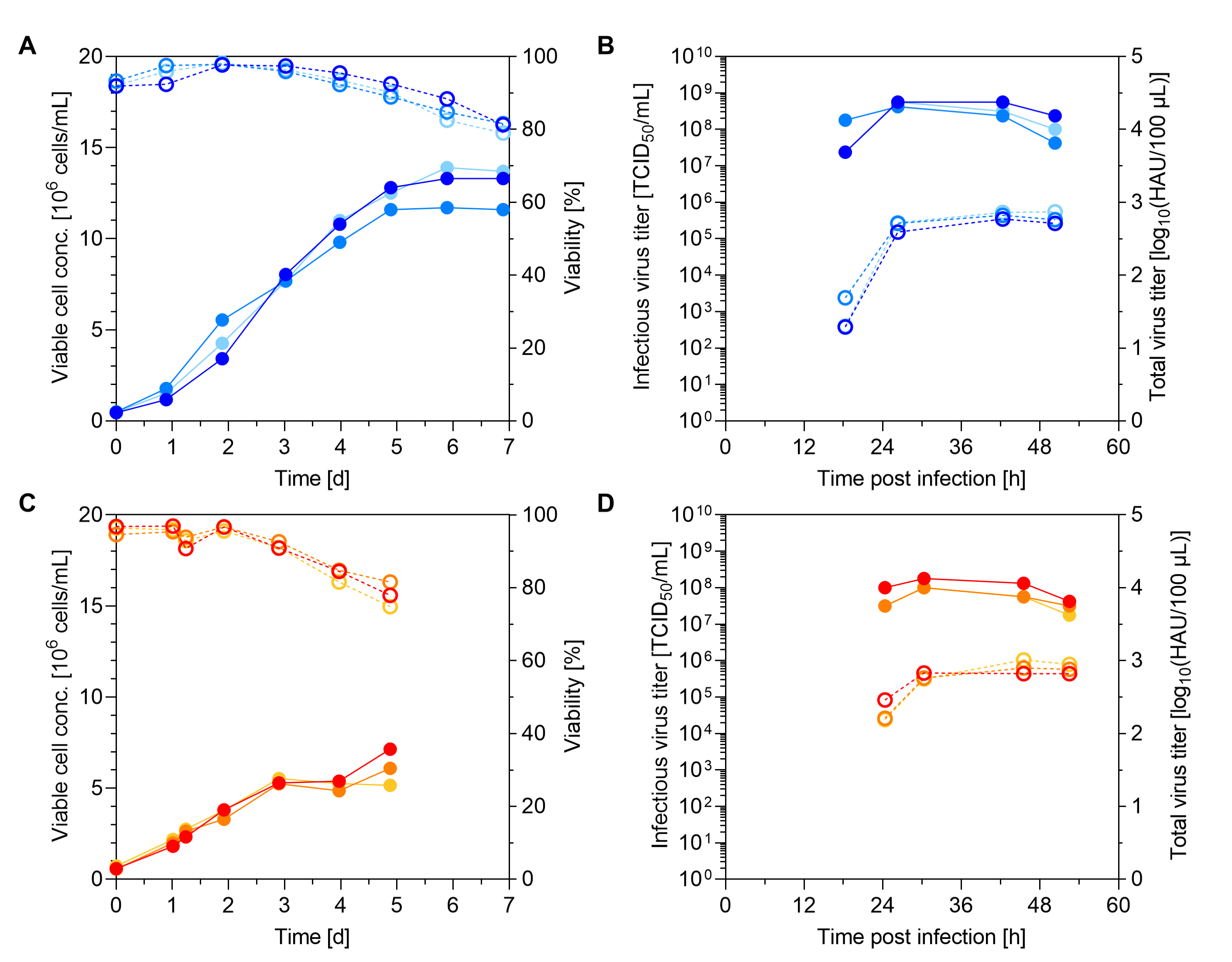


Supplementary Figure 1. Assessment of Growth and Infection Stability of Clonal MDCK Cultures in Non-Baffled Shake Flasks. Cells were thawed and research cell banks were generated after 7, 14, and 21 passages. The resulting cell banks were simultaneously tested with respect for cell growth and virus production. Viable cell concentration and viability were monitored over 5 to 7 days of growth in batch mode in 30 mL MDXK medium (A: C59, C: C113). Infection of cells with IAV using 3% TrypLE10X (B: C59, D: C113). Infectious virus titers were determined by TCID_50_ assay and total virus titers by HA assay. Full circles with solid lines represent the left y-axis, while empty circles with dashed lines represent the right y-axis.


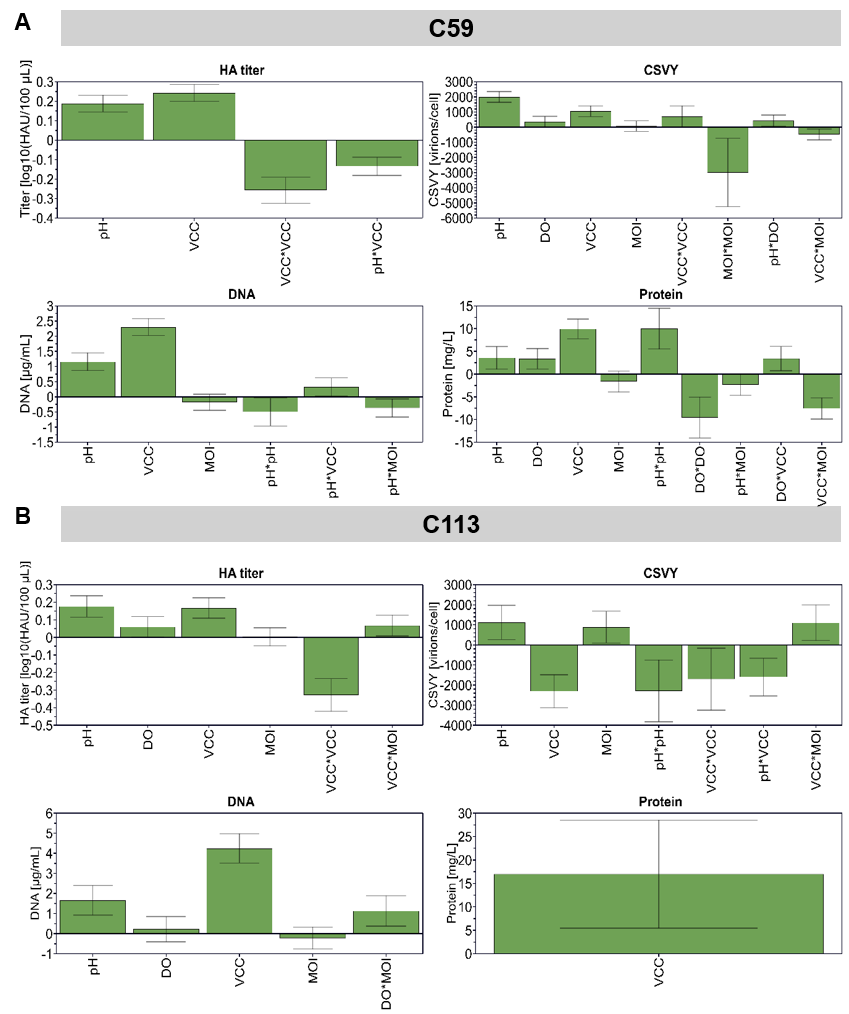


Supplementary Figure 2. Coefficient Plot of the Critical Process Parameters. The influence of the CPPs on the target response variables is shown for C59 (A) and C113 (B). Positive and negative coefficients indicate the direction of the effect, while error bars represent the confidence intervals, highlighting the reliability of the estimate for each parameter. Insignificant coefficients were excluded except for interaction terms (p-value < 0.05).


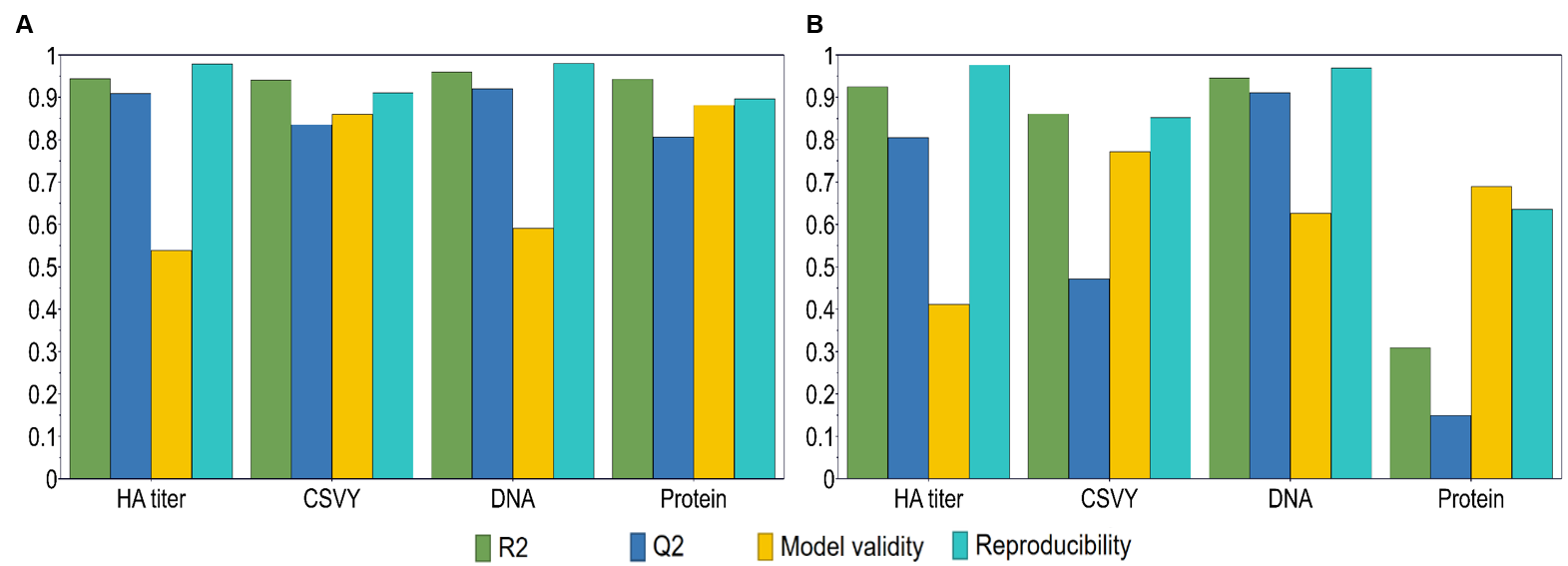


Supplementary Figure 3. Model Statistics for the Critical Quality Attributes. Various statistical metrics were evaluated to assess the fit and applicability of the model for C59 (A) and C113 (B).
